# Supplementary material for: Specific exercise patterns generate an epigenetic molecular memory window that drives long-term memory formation and identifies ACVR1C as a bidirectional regulator of memory in mice
Source: Nat Commun. 2024 May 7;15:3836. doi: 10.1038/s41467-024-47996-w (PMC11076285; doi:10.1038/s41467-024-47996-w)
Supplement: Supplementary file 1 — Supplementary Information [file 41467_2024_47996_MOESM1_ESM.pdf]

Supplemental Information

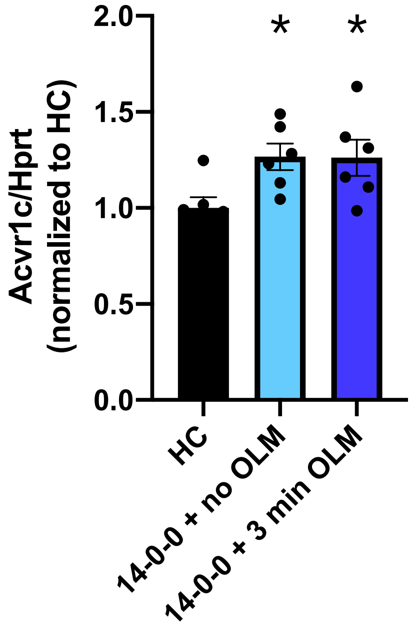

Figure S1. Exercise alone modulates *Acvr1c* mRNA levels in dorsal hippocampus. Independent 12-week-old mouse cohort (n=6 male mice/group) undergoing 14 days of exercise (14-0-0) and receiving either subthreshold 3 -minute object location memory (OLM) training or no training (home cage/HC). RT-qPCR data demonstrating greater *Acvr1c* mRNA abundance in dorsal hippocampus following 2-week exercise prior to training and in previous conditions (Figure 2D) where exercise facilitates learning relative to home cage controls. One-way ANOVA, Group ($F_{(2,15)}$ = 4.22, P = 0.035). Dunnett's post hoc test: *P <0.05, compared to home cage control. Normalization to home cage control. Data are presented as mean ± SEM. Source data are provided as a Source Data file.

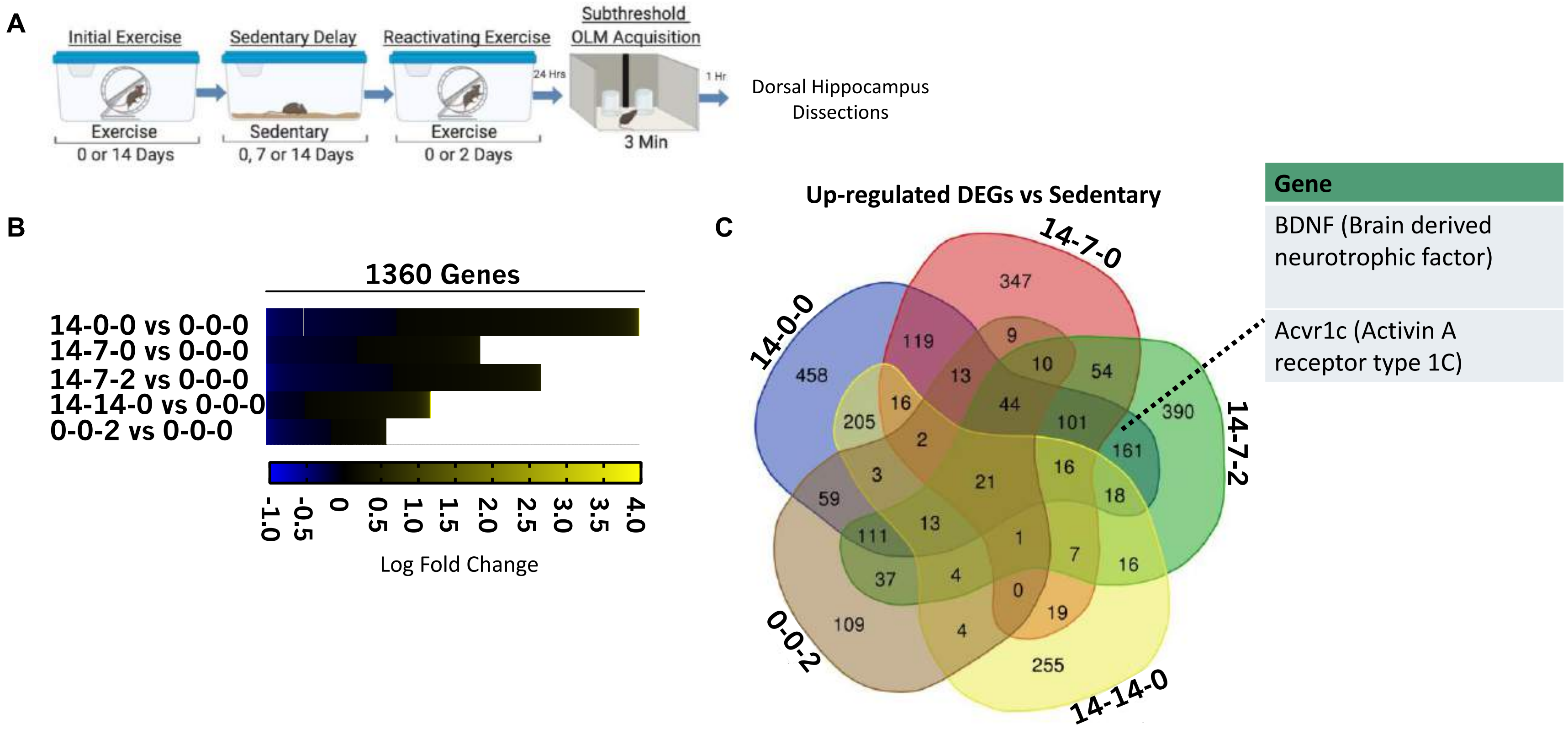

Figure S2. *Acvr1c* expression in hippocampus is induced during memory consolidation only under exercise conditions that facilitate memory encoding: linear regression approach. (A) Schematic of different exercise regimes prior to object location memory (OLM). Created with Biorender.com. (B) Heat map of genes differentially expressed in each condition compared to sedentary control (0-0-0) utilizing linear regression approach. Positive log fold change indicates up-regulated genes vs sedentary controls whereas negative log fold change refers to down-regulated genes. (C) Number of up-regulated differentially expressed genes (DEGs) in dorsal hippocampus compared to sedentary utilizing linear regression approach. *Acvr1c* and *Bdnf* are also up-regulated utilizing a linear regression model (as in Figure 2 utilizing Bayesian-regularized *t*-test model) in dorsal hippocampus only in conditions where exercise enabled learning under inadequate, subthreshold conditions (14-0-0 and 14-7-2).

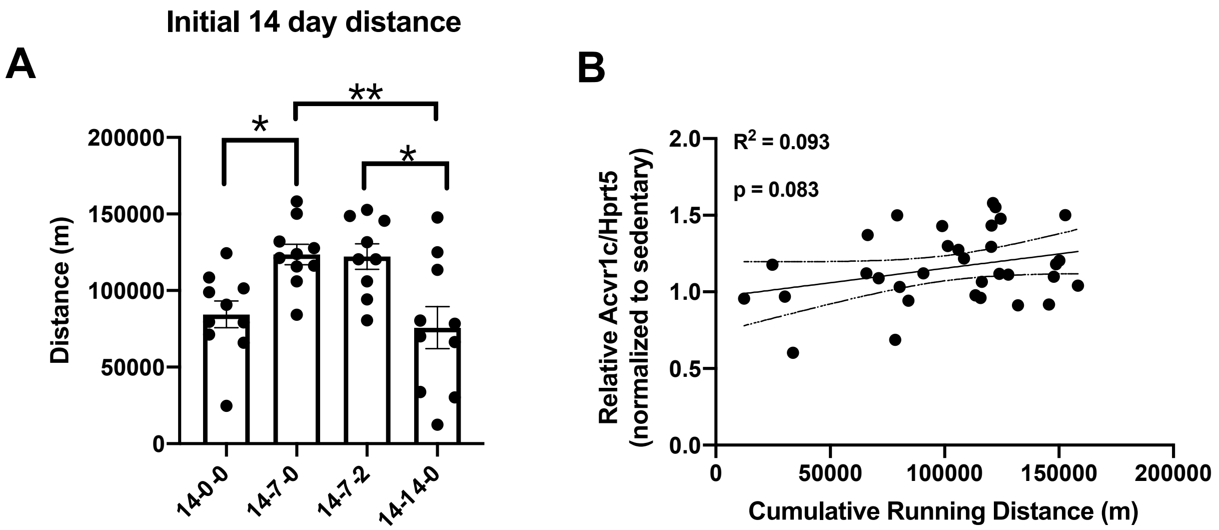

Figure S3. Running distance for initial 14-day period in each group used in RNA-sequencing studies. (A) Cumulative distance run by mice in specific exercise groups utilized in RNA-Seq for initial 14-day period. One-way ANOVA revealed a main effect of Group: ($F_{(3,35)}$ = 6.46, P <0.001). Tukey's post hoc test: *P <0.05, **P <0.01, (14-0-0: n=10, 14-7-0: n=10, 14-7-2: n=9, 14-14-0: n=10). Data are presented as mean ± SEM. (B) The overall regression was not statistically significant (R2=0.093, F (1,31)=3.209, p=0.083). The results of the regression indicated running distance explained 9.3% of the variation in relative *Acvr1c/Hprt* mRNA levels. Source data are provided as a Source Data file.

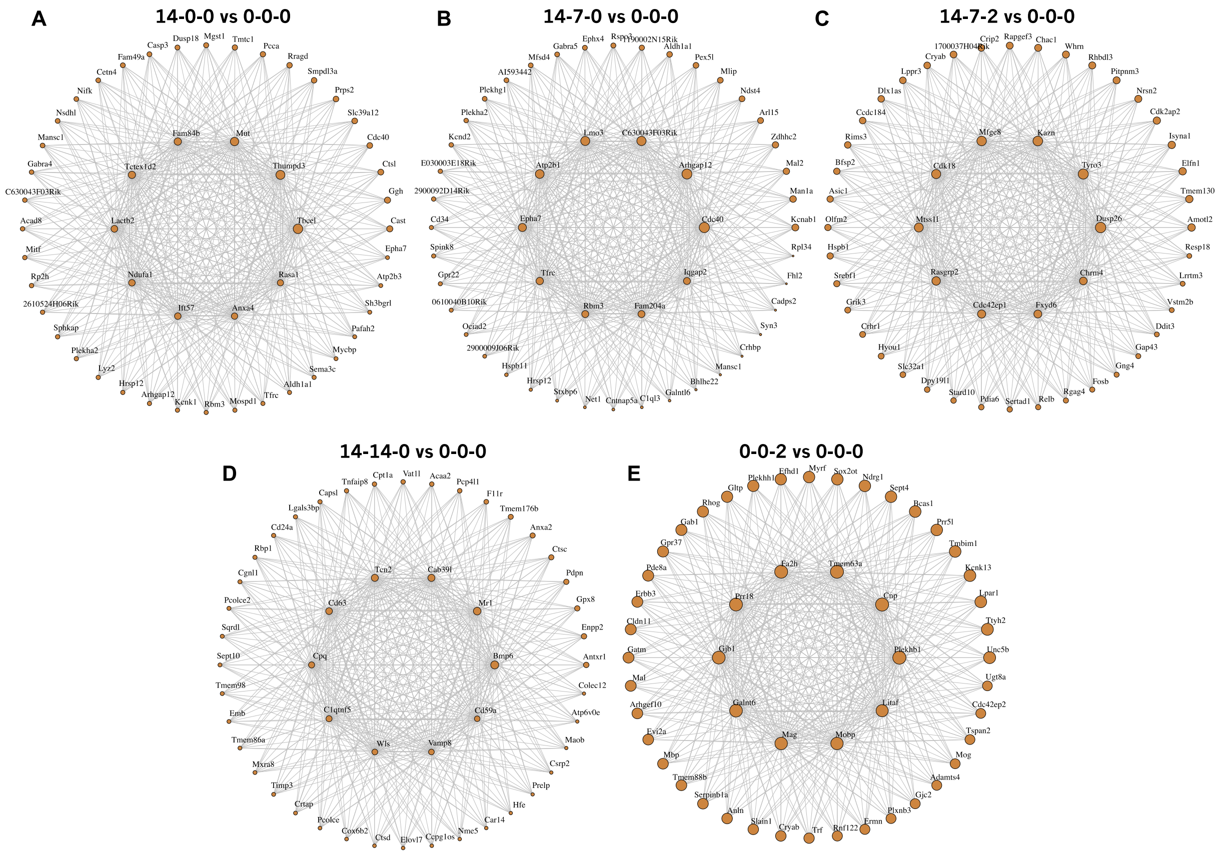

Figure S4. Gene coexpression network analysis. Gene coexpression network plot showing hub genes in the center. Comparisons refer to up-regulated genes compared with sedentary control for exercise groups 14-0-0 (A), 14-7-0 (B), 14-7-2 (C), 14-14-0 (D), 0-0-2 (E) in Figure 2.

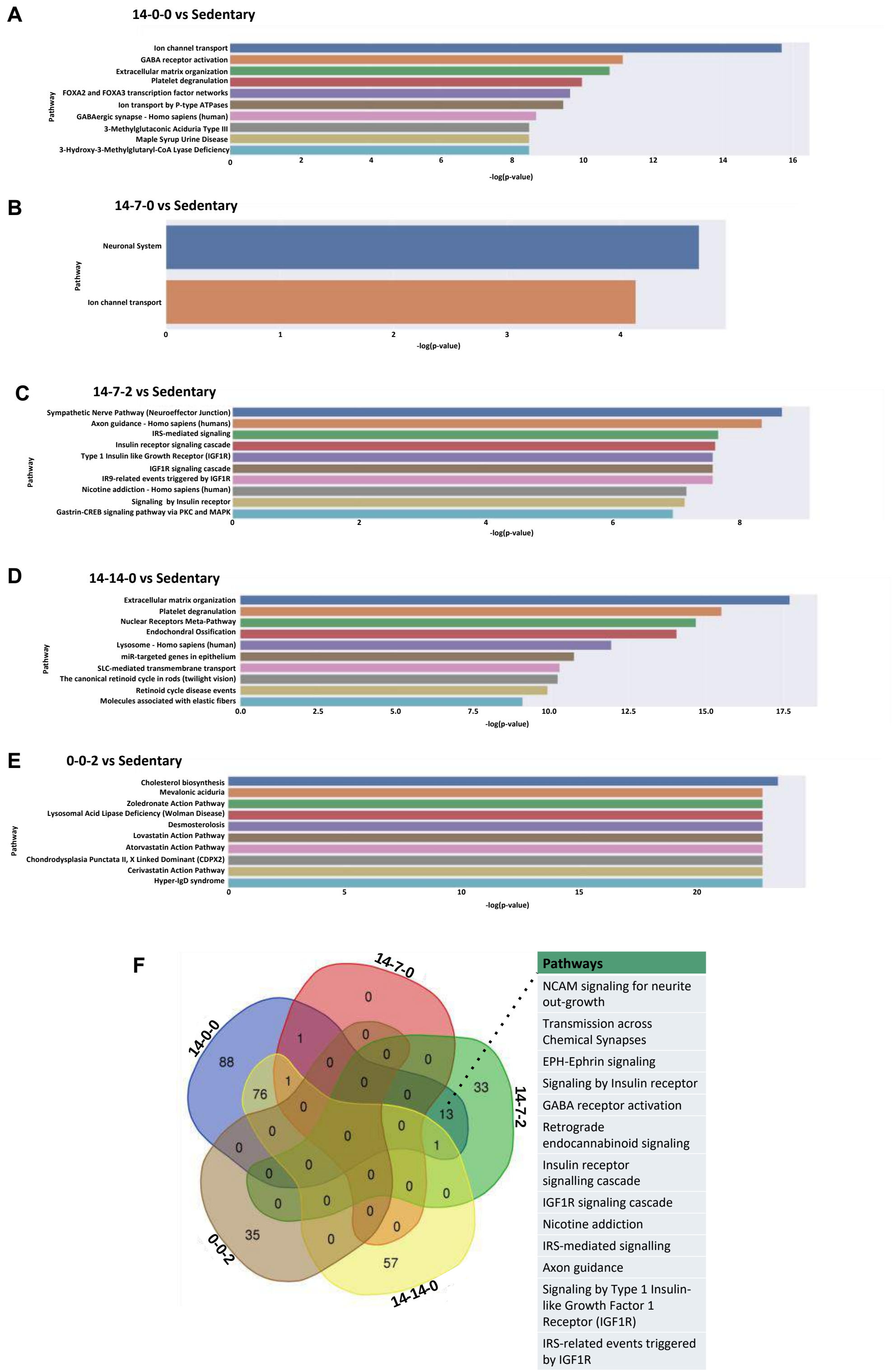

Figure S5. Predicted pathways altered by exercise. Top pathways of up-regulated DEGs compared with sedentary control for exercise groups 14-0-0 (A), 14-7-0 (B), 14-7-2 (C), 14-14-0 (D), 0-0-2 (E). (F) Number of top predicted pathways of up-regulated DEGs compared with sedentary control in Figure 2. Pathways from up-regulated DEGs in dorsal hippocampus only in conditions where exercise facilitates learning are displayed to the right.

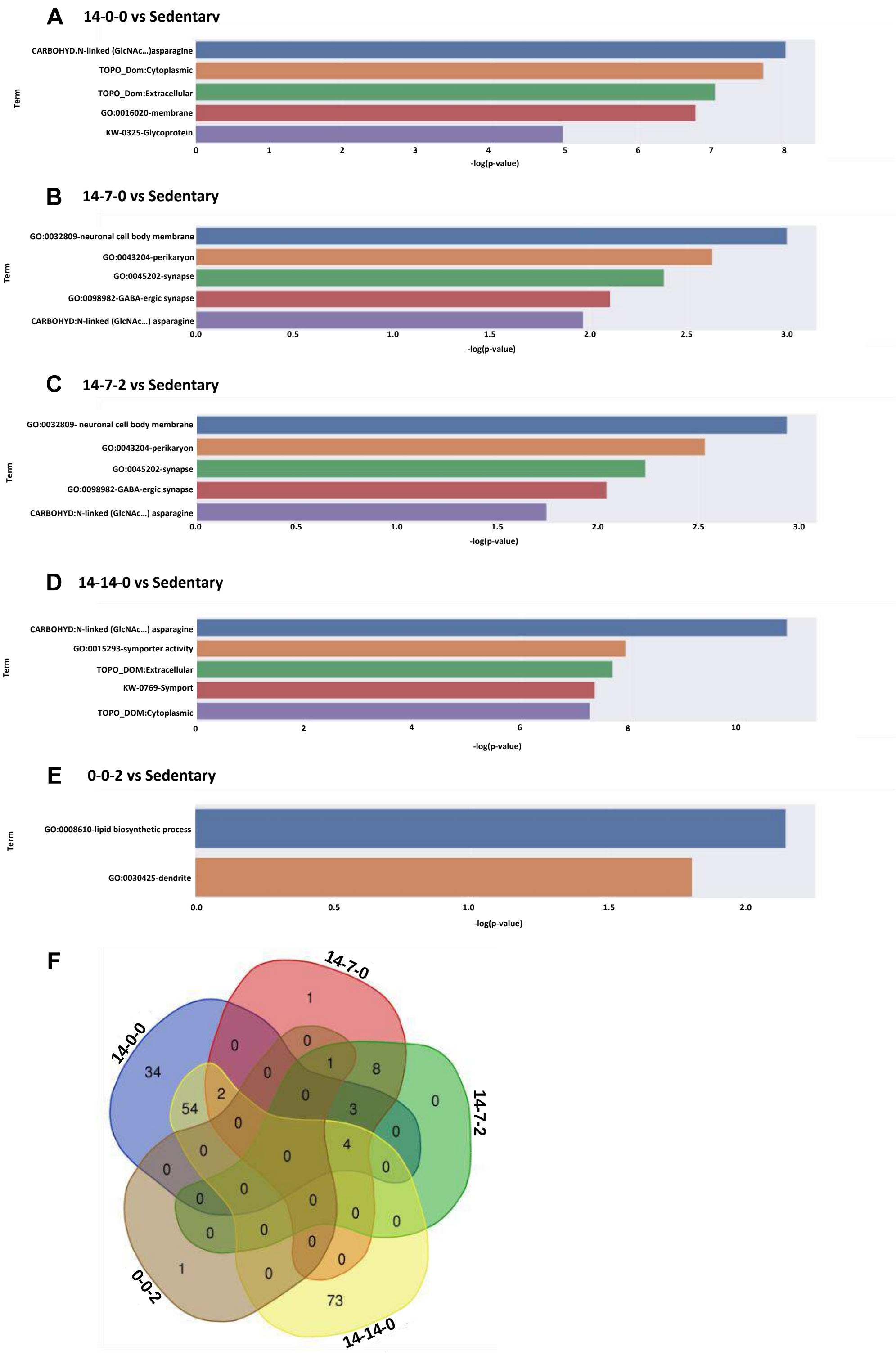

Figure S6. Predicted gene ontology (GO) enrichment analysis of cellular components altered by exercise. Top biological processes of up-regulated DEGs compared with sedentary control for exercise groups 14-0-0 (A), 14-7-0 (B), 14-7-2 (C), 14-14-0 (D), 0-0-2 (E). (F) Number of top GO terms of up-regulated DEGs compared with sedentary control. GO terms from up-regulated DEGs in dorsal hippocampus only in conditions where exercise facilitates learning are displayed to the right.

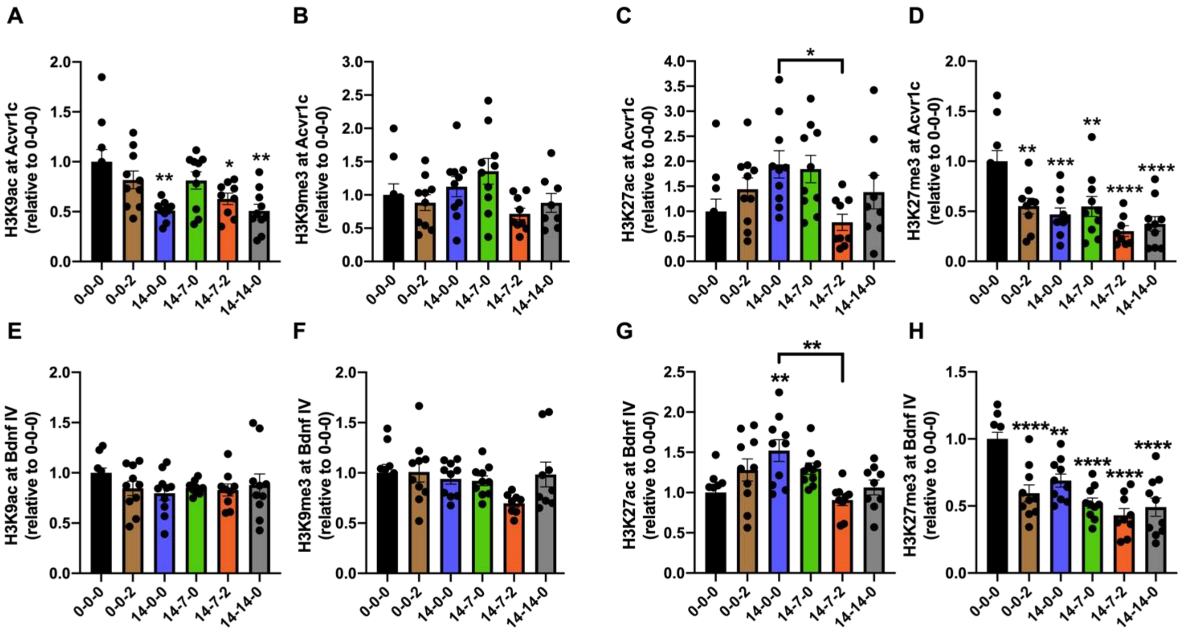

Figure S7. Exercise modulates epigenetic regulation of *Acvr1c* and *Bdnf* during consolidation and reveals a specific permissive signature. (A-D) Exercise does not impact (A) H3K9ac following sedentary delay periods: One-way ANOVA, Group ($F_{(5,52)}$ = 5.68, P = 0.0003), (0-0-0: n=10, 0-0-2: n=10, 14-0-0: n=9, 14-7-0: n =10, 14-7-2: n=9, 14-14-0: n=10). (B) H3K9me3: One-way ANOVA, Group ($F_{(5,50)}$ = 2.31, P = 0.0572), (0-0-0: n=9, 0-0-2: n=10, 14-0-0: n=10, 14-7-0: n =10, 14-7-2: n=9, 14-14-0: n=8) or (C) H3K27ac: One-way ANOVA, Group ($F_{(5,52)}$ = 3.07, P = 0.0167), (0-0-0: n=10, 0-0-2: n=10, 14-0-0: n=10, 14-7-0: n =10, 14-7-2: n=9, 14-14-0: n=9) occupancy at the *Acvr1c* promoter. (D) Minimal (2-day), (**P <0.01) or extensive (14-day), (***P <0.001) exercise remarkably reduces repressive H3K27me3 at the *Acvr1c* promoter. Notably, this reduction continues at the sedentary delay periods (7-day), (**P <0.01) and (14-day), (****P <0.0001) and following reactivating exercise (****P <0.0001): (One-way ANOVA, Group ($F_{(5,51)}$ = 8.54, P < 0.0001), (0-0-0: n=10, 0-0-2: n=9, 14-0-0: n=10, 14-7-0: n =10, 14-7-2: n=8, 14-14-0: n=10). Exercise does not impact (E) H3K9ac: One-way ANOVA, Group ($F_{(5,53)}$ = 1.06, P = 0.3893), (0-0-0: n=10, 0-0-2: n=10, 14-0-0: n=10, 14-7-0: n =10, 14-7-2: n=9, 14-14-0: n=10). (F) H3K9me3: One-way ANOVA, Group ($F_{(5,51)}$ = 1.85, P = 0.1181), (0-0-0: n=10, 0-0-2: n=10, 14-0-0: n=10, 14-7-0: n =10, 14-7-2: n=8, 14-14-0: n=9) or (G) H3K27ac: One-way ANOVA, Group ($F_{(5,52)}$ = 4.98, P = 0.0008), (0-0-0: n=10, 0-0-2: n=10, 14-0-0: n=10, 14-7-0: n =10, 14-7-2: n=9, 14-14-0: n=9) occupancy at the *Bdnf IV* promoter. (H) Remarkably, engagement in either minimal (2-day), (****P <0.0001) or extensive (14-day) (**P <0.01) exercise also reduces repressive H3K27me3 at the *Bdnf IV* promoter. Notably, this reduction also continues at the sedentary delay periods assessed (7-day), (****P <0.0001) and (14-day), (****P <0.0001) sedentary. Source data are provided as a Source Data file.

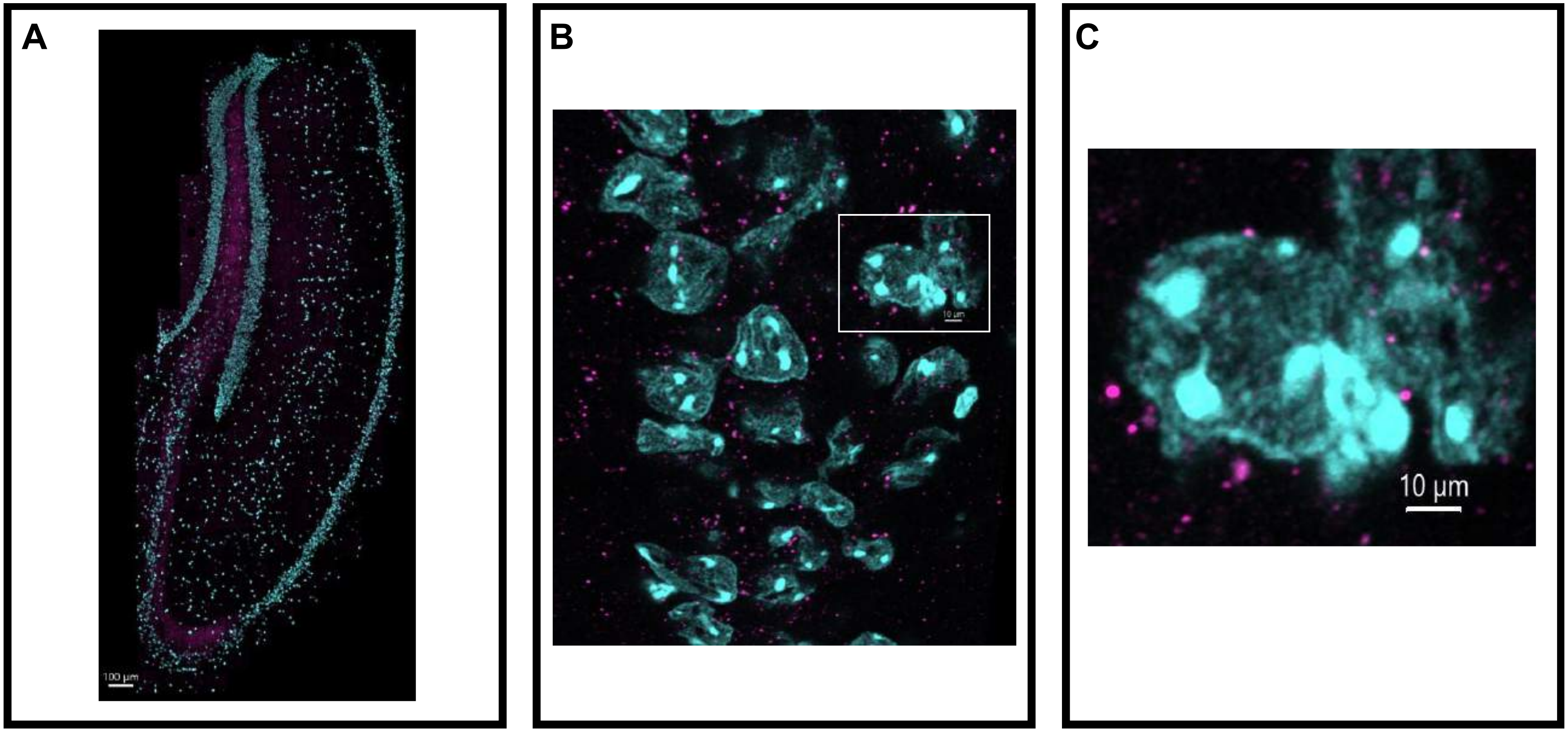

Figure S8. ACVR1C spread in dorsal hippocampus. (A) Immunofluorescent image of ACVR1C expression in the dorsal hippocampus of a young, 3-month-old male mouse. (B) ACVR1C expression in area CA1 of the dorsal hippocampus. (C) 60x magnification outcropping from panel B (white box) displaying ACVR1C expression in CA1. Source data are provided as a Source Data file.

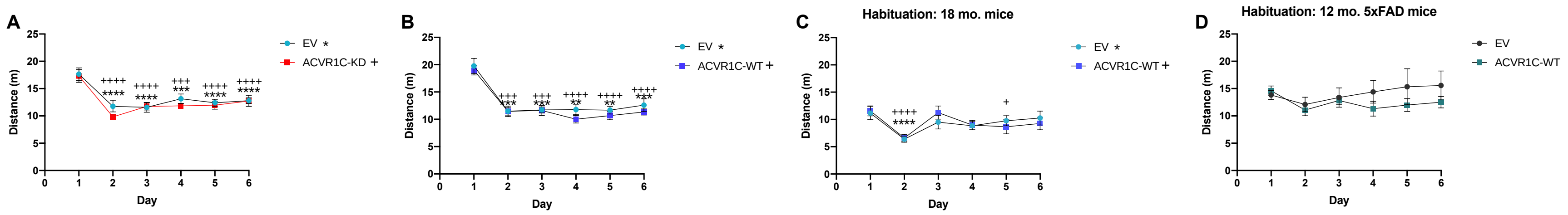

Figure S9. *Acvr1c* manipulation has no effect on movement and all groups habituate to the context prior to OLM training. (A) Total distance traveled during each day of OLM habituation for mice in Figure 4. Mice from both groups habituate to the context (Repeated measures Two-way ANOVA: Day: (5,85)=22.88, P < 0.0001). Group differences in distance travelled are not observed Group: (1,17)=0.70, P = 0.414), Interaction: (5,85)=0.66, P = 0.653); Tukey's post hoc test: ***P <0.001, ****P <0.0001, (empty vector (EV): n=9, ACVR1C-kinase dead (ACVR1C-KD): n=10). (B) Total distance traveled during each day of OLM habituation for mice in Figure 5. Mice from both groups habituate to the context (Repeated measures Two-way ANOVA: Day: (3.22, 58)=45.45, P < 0.0001). Group differences in distance travelled were not observed Group: (1,18)=0.803, P = 0.382), Interaction: (5,90)=0.482, P = 0.788); Tukey's post hoc test: **P <0.01, ***P <0.001, ****P <0.0001, (EV: n=11, ACVR1C wild type (ACVR1C-WT): n=9). (C) Total distance traveled during each day of OLM habituation for 18 mo. C57 mice in Figure 6. Mice from both groups habituate to the context as indicated by lower exploration on day 2 relative to day 1 for the EV group and lower exploration on day 2 and 5 in the ACVR1C-WT mice (Repeated measures Two-way ANOVA: Day: (5,60)=11.70, P < 0.0001). Group differences in distance travelled were not observed Group: (1,12)=0.003, P = 0.951), Interaction: (5,60)=1.17, P = 0.332); Tukey's post hoc test: *P <0.05, ****P <0.0001, (EV: n=7, ACVR1C-WT: n=7). (D) Total distance traveled during each day of OLM habituation for 12 mo. 5xFAD mice in Figure 6. Mice from both groups show no change in distance travelled across habituation days (Repeated measures Two-way ANOVA: Day: (5,105)=1.15, P = 0.18). Group differences in distance travelled were not observed Group: (1,21)=0.99, P = 0.32), Interaction: (5,105)=1.17, P = 0.32), (EV: n=10, ACVR1C-WT: n=13). Data are presented as mean ± SEM. Source data are provided as a Source Data file.

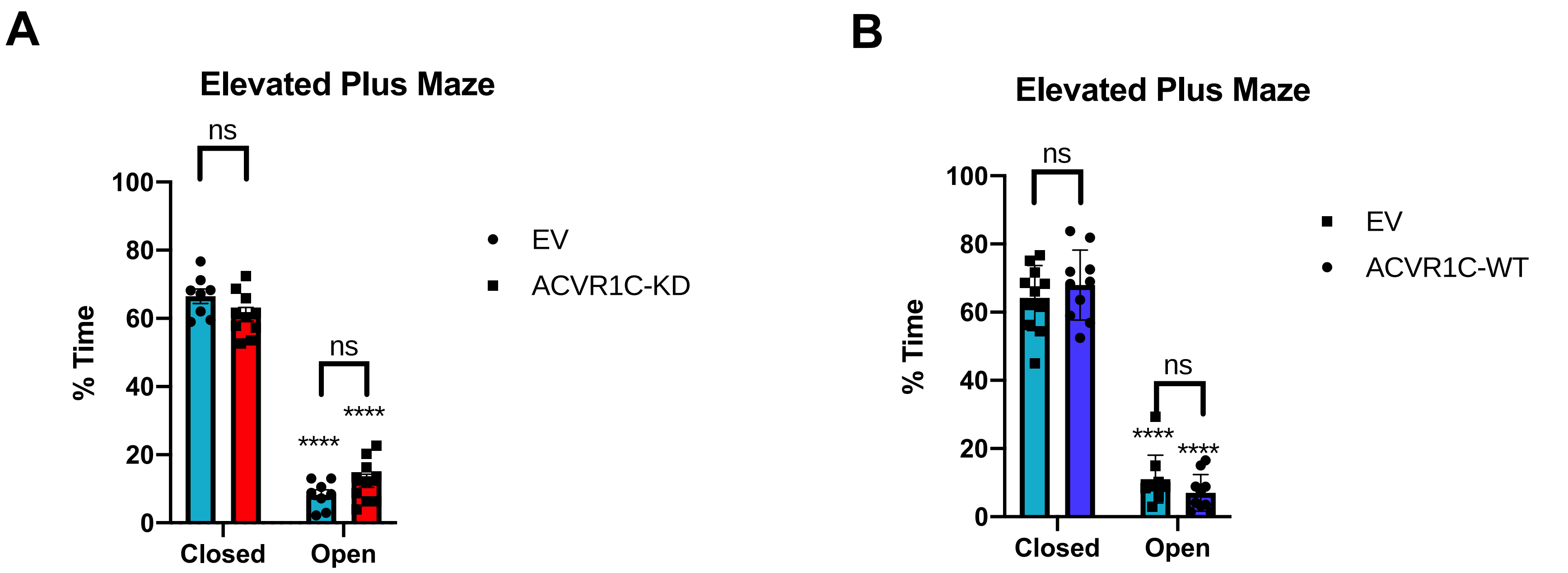

Figure S10. *Acvr1c* manipulation has no effect on anxiety. (A) Animals from Figure 3 were tested in the elevated plus maze. Both groups show similar levels of anxiety, as indicated by the percentage of time spent in the closed and open arms (Repeated measures two-way ANOVA: Group: (1,17)= 0.377, P = 0.546), Arm: (1,17)= 529.6, P < 0.0001), Interaction: (1,17)= 5.010, P = 0.038); (empty vector (EV): n=8, ACVR1C kinase-dead (ACVR1C-KD): n=10). Sidak post hoc test: ****P <0.0001 compared with closed. (B) Animals from Figure 4 were tested in the elevated plus maze. Both groups show similar levels of anxiety, as indicated by the percentage of time spent in the closed and open arms (Repeated measures two-way ANOVA: Group: (1,38)= 0.002, P = 0.956), Arm: (1,38)= 497.4, P < 0.0001), Interaction: (1,38)= 2.296, P = 0.138); (EV: n=11, ACVR1C wild type (ACVR1C-WT): n=10). Sidak post hoc test: ****P <0.0001 compared with closed. Data are presented as mean ± SEM. Source data are provided as a Source Data file.

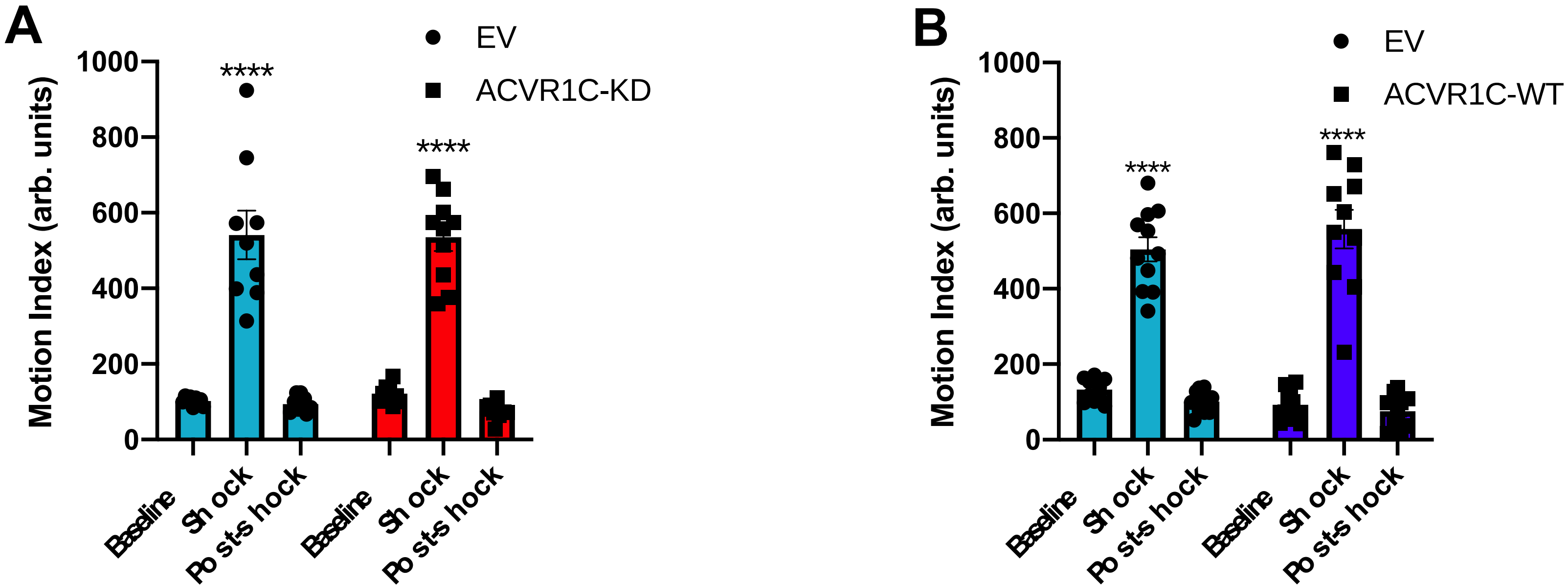

Figure S11. *Acvr1c* manipulation has no effect on shock reactivity. (A) Groups from Figure 4 are similarly reactive to shock onset during fear conditioning (Two-way ANOVA: Bin: (2,34)= 135.1, P < 0.0001), Group: (1,17)= 0.013, P = 0.908), Interaction: (2,34)= 0.167, P = 0.846); (empty vector (EV): n=9, ACVR1C kinase-dead (ACVR1C-KD): n=10); Tukey's post hoc test: ****P <0.0001. (B) Groups from Figure 5 are similarly reactive to shock onset during fear conditioning (Two-way ANOVA: Bin: (2,38)= 195.9, P < 0.0001), Group: (1,19)= 0.030, P = 0.863), Interaction: (2, 38)= 1.997, P = 0.149); (EV: n=11, ACVR1C-WT: n=10); Tukey's post hoc test: ****P <0.0001. Baseline refers to the motion occurring before the shock, Shock refers to the motion during the 2 second footshock, and post-shock refers to the motion during the 30 seconds following the shock. Data are presented as mean ± SEM. Source data are provided as a Source Data file.

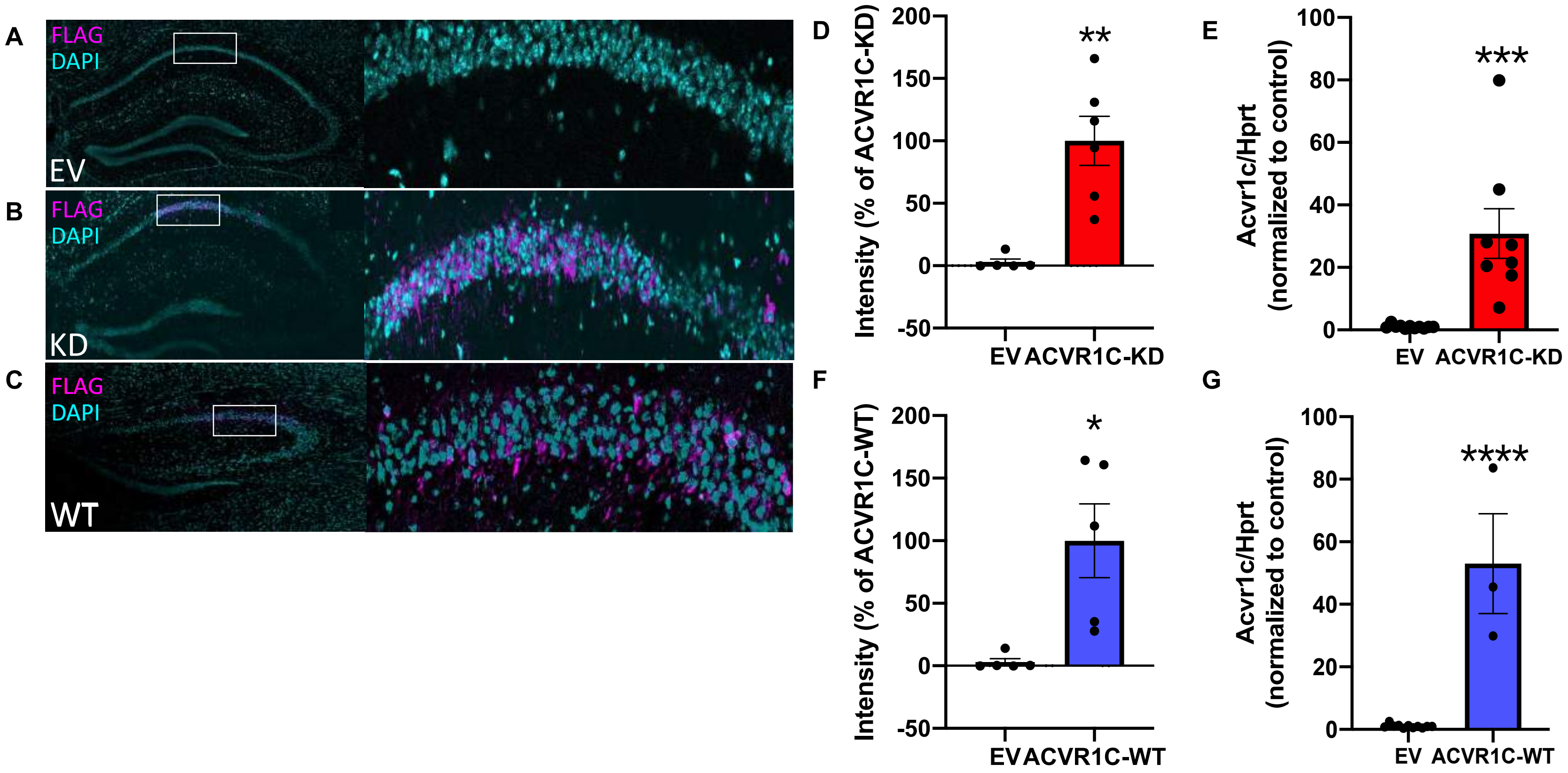

Figure S12. *Acvr1c* disruption or overexpression in the dorsal hippocampus. (A) Representative immunofluorescence images of FLAG (magenta) expression in adult mice injected with control virus (AAV1-CMV-V5), (B) *Acvr1c* kinase-dead mutant construct (AAV1-CMV-mAc-3flag or (C) overexpressing *Acvr1c* (AAV1-CMV-mAcvr1c-3flag). Cell bodies were counterstained with DAPI (teal). (D) Mean intensity of FLAG immunofluorescence sampled from CA1 (normalized to background). FLAG expression was significantly enhanced in mice overexpressing the *Acvr1c* kinase-dead construct compared to AAV-EV controls independent two-sample *t*-test (two-tailed): (t(9)=4.459, P =0.001), (empty vector (EV): n=5, ACVR1C kinase-dead (ACVR1C-KD): n=6). (E) *Acvr1c* mRNA expression in the dorsal hippocampus. *Acvr1c* was significantly enhanced in mice overexpressing the *Acvr1c* kinase-dead construct compared to AAV-EV controls independent two-sample *t*-test (two-tailed): (t(17)=4.433, P =0.0004), (EV: n=11, ACVR1C-KD: n=8). (F) FLAG expression was significantly enhanced in mice overexpressing the wildtype Acvr1c construct compared to AAV-EV controls independent two-sample *t*-test (two-tailed): (t(8)=3.282, P =0.011), (EV: n=5, ACVR1C- wild type (ACVR1C-WT): n=5). (G) *Acvr1c* mRNA expression in the dorsal hippocampus. *Acvr1c* was significantly enhanced in mice overexpressing the wildtype *Acvr1c* construct compared to AAV-EV controls independent two-sample *t*-test (two-tailed): (t(12)=7.071, P =0.0001), (EV: n=11, ACVR1C-WT: n=3). *P <0.05, **P <0.01, ***P <0.001, ****P <0.0001. Data are presented as mean ± SEM. Source data are provided as a Source Data file.

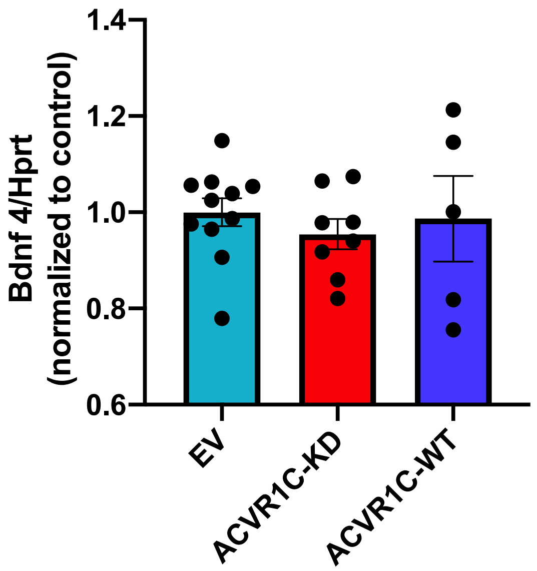

Figure S13. ACVR1C manipulation does not impact *Bdnf IV* mRNA levels. RT-qPCR data demonstrating no change in *Bdnf IV* in dorsal hippocampus following overexpression with the ACVR1C kinase dead (ACVR1C-KD) or wildtype (ACVR1C-WT) constructs One-way ANOVA, Group ($F_{(2,21)}$= 0.33, P = 0.721), (EV: n=11, ACVR1C-KD: n=8, ACVR1C-WT: n=5). Data are presented as mean ± SEM. Source data are provided as a Source Data file.

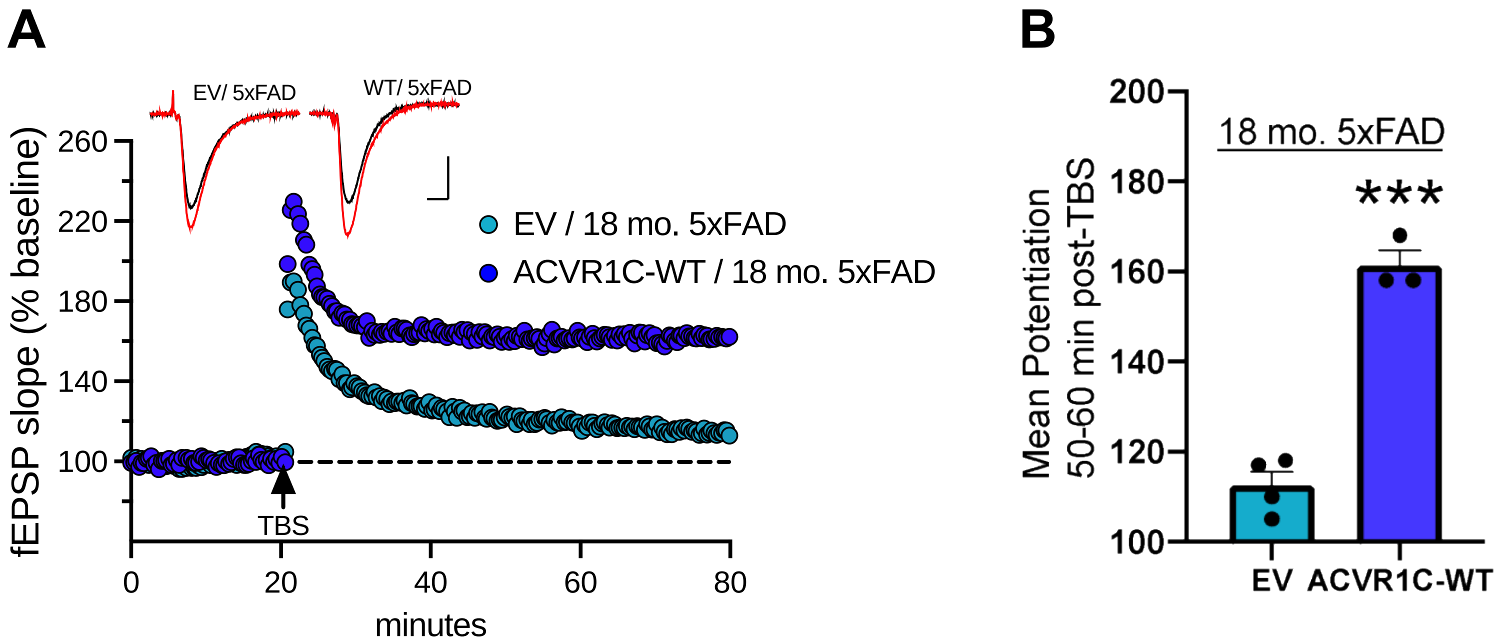

Figure S14. Age and AD-related impairments in hippocampus-dependent synaptic plasticity are ameliorated by overexpressing ACVR1C. (A) LTP as mean ± SEM fEPSP slope as percentage of baseline overtime (ACVR1C- wild type (ACVR1C-WT)/5xFAD; n=3 mice,6 slices, empty vector (EV)/5xFAD; n=4 mice, 8 slices). Inset; representative traces collected during baseline (black line) and 60 min post-theta burst stimulation (TBS) (red line) from ACVR1C-WT/5xFAD and EV/5xFAD group. Scale = 1mV/5ms. (B) Mean ± SEM level of potentiation 50-60 min post TBS showing ACVR1C overexpression enhances LTP in slices from 18 mo. 5xFAD mice relative to EV control independent two-sample *t*-test (two-tailed): (t(5)=10.67, P =0.0001). ***P <0.001. Source data are provided as a Source Data file.

DEGs Up-regulated during consolidation only under conditions where exercise facilated memory formation (14-0-0 and 14-7-2) vs. Sedentary control: (Bayesian-regularized t-test model)						
**Gene**	14-0-0 **(Mean)**	Sed **(Mean)**	Fold change **(Log)**	14-7-2 **(Mean)**	Sed **(Mean)**	Fold change **(Log)**
Neurod6	165.9588	149.506565	0.10439926	165.2967	149.50657	0.100401736
Ppp1r1a	72.73144	59.13207	0.207000336	77.43649	59.13207	0.2696847
Cnksr2	66.44482	57.740174	0.140418641	67.90284	57.740174	0.162124672
Btbd3	105.57756	88.91967	0.171712471	107.30188	88.91967	0.187912793
Necab3	13.48469	10.884601	0.21420593	14.20219	10.884601	0.26604714
Rragd	32.91871	27.17786	0.191638518	33.00002	27.17786	0.194105496
Gabra4	40.97199	34.44417	0.17354891	41.47379	34.44417	0.185721909
Npy1r	15.84094	13.284606	0.175991807	16.509679	13.284606	0.217340894
Ano3	45.62066	38.223216	0.176917604	44.27575	38.223216	0.146994043
Rasgrp1	211.7399	186.27571	0.128130747	218.3022	186.27571	0.158652453
Kcnj3	49.64485	42.00452	0.167117427	49.76588	42.00452	0.169552377
Epha4	112.7867	95.79207	0.163318519	115.9606	95.79207	0.191070573
Acvr1c	4.856885	3.451439	0.34160604	4.84239	3.451439	0.338617155
Fgf13	146.1576	122.81474	0.17400845	150.4445	122.81474	0.202917204
Robo2	17.88978	15.16782	0.165053522	17.81961	15.16782	0.161123458
Sprn	101.24211	85.57845	0.168081278	103.65588	85.57845	0.191643068
Synpr	102.36603	83.57193	0.202847221	99.8834	83.57193	0.178295808
Zbtb18	257.2488	222.53637	0.144953161	260.5942	222.53637	0.157873859
Bdnf	23.77175	18.205906	0.266751854	21.66434	18.205906	0.173921544
Rasa1	22.63607	18.620784	0.195266275	22.24507	18.620784	0.177842035
Fam49a	75.1823	64.17484	0.158304597	74.25388	64.17484	0.145878799

Table S1. Table containing the full list of Up-regulated differentially expressed genes (DEGs) in dorsal hippocampus during consolidation only under conditions when exercise facilitated memory formation (14-0-0 and 14-7-2) vs sedentary control utilizing the Bayesian-regulated t-test model.

Alignment Rate	Sample	Total Reads
95.00%	L1-P1-CGATGT	40753418
95.70%	L1-P2-TGACCA	40074824
95.30%	L1-P3-ATGTCA	53028705
95.50%	L1-P4-ACAGTG	36476189
94.50%	L1-P5-GCCAAT	34408369
95.90%	L1-P6-CAGATC	49603649
95.50%	L1-P7-CTTGTA	24322516
94.50%	L1-P8-AGTCAA	45743178
95.40%	L1-P9-AGTTCC	43099144
87.30%	L2-P1-CGATGT	45182354
89.60%	L2-P2-AGTTCC	47897099
90.90%	L2-P3-TGACCA	48138470
88.40%	L2-P4-ACAGTG	20104166
91.30%	L2-P5-GCCAAT	35679348
91.40%	L2-P6-CAGATC	46982779
93.30%	L2-P7-CTTGTA	47681468
94.00%	L2-P8-AGTCAA	39159605
95.00%	L2-P9-ATGTCA	39393770
88.60%	L3-P1-AGTCAA	40643499
93.00%	L3-P2-AGTTCC	37308498
85.90%	L3-P3-CGATGT	48144379
93.80%	L3-P4-TGACCA	39448761
92.90%	L3-P5-ACAGTG	41796466
89.00%	L3-P6-GCCAAT	49344860
93.70%	L3-P7-CAGATC	47906905
94.00%	L3-P8-ATGTCA	33280147
90.10%	L3-P9-CTTGTA	35201528
92.60%	L4-P1-CTTGTA	30993955
87.90%	L4-P2-AGTCAA	40496994
88.60%	L4-P3-CGATGT	51086283
89.80%	L4-P4-AGTTCC	76603517
91.90%	L4-P5-TGACCA	45351292
88.70%	L4-P6-ACAGTG	1024840
92.30%	L4-P7-GCCAAT	61907597
91.20%	L4-P8-ATGTCA	31986059
90.70%	L4-P9-CAGATC	30862836
95.00%	L5-P1-CAGATC	36904459
94.90%	L5-P2-CTTGTA	41166334
95.00%	L5-P3-AGTCAA	35158289
95.30%	L5-P4-AGTTCC	21229393
94.70%	L5-P5-CGATGT	47975500
94.30%	L5-P6-TGACCA	44426989

Table S2. Table containing the number of reads and alignment rate for each sequencing sample.
