# Supplementary material for: Specific exercise patterns generate an epigenetic molecular memory window that drives long-term memory formation and identifies ACVR1C as a bidirectional regulator of memory in mice
Source: Nat Commun. 2024 May 7;15:3836. doi: 10.1038/s41467-024-47996-w (PMC11076285; doi:10.1038/s41467-024-47996-w)
Supplement: Supplementary file 3 — Reporting Summary [file 41467_2024_47996_MOESM3_ESM.pdf]

nature portfolio

Corresponding author(s): Marcelo Wood

Last updated by author(s): Mar 22, 2024

Reporting Summary

Nature Portfolio wishes to improve the reproducibility of the work that we publish. This form provides structure for consistency and transparency in reporting. For further information on Nature Portfolio policies, see our Editorial Policies and the Editorial Policy Checklist.

Statistics

For all statistical analyses, confirm that the following items are present in the figure legend, table legend, main text, or Methods section.

n/a	Confirmed	
☐	☒	The exact sample size (*n*) for each experimental group/condition, given as a discrete number and unit of measurement
☐	☒	A statement on whether measurements were taken from distinct samples or whether the same sample was measured repeatedly
☐	☒	The statistical test(s) used AND whether they are one- or two-sided<br>*Only common tests should be described solely by name; describe more complex techniques in the Methods section.*
☐	☒	A description of all covariates tested
☐	☒	A description of any assumptions or corrections, such as tests of normality and adjustment for multiple comparisons
☐	☒	A full description of the statistical parameters including central tendency (e.g. means) or other basic estimates (e.g. regression coefficient) AND variation (e.g. standard deviation) or associated estimates of uncertainty (e.g. confidence intervals)
☐	☒	For null hypothesis testing, the test statistic (e.g. *F*, *t*, *r*) with confidence intervals, effect sizes, degrees of freedom and *P* value noted<br>*Give P values as exact values whenever suitable.*
☒	☐	For Bayesian analysis, information on the choice of priors and Markov chain Monte Carlo settings
☒	☐	For hierarchical and complex designs, identification of the appropriate level for tests and full reporting of outcomes
☒	☐	Estimates of effect sizes (e.g. Cohen's *d*, Pearson's *r*), indicating how they were calculated

Our web collection on statistics for biologists contains articles on many of the points above.

Software and code

Policy information about availability of computer code

Data collection	All data were collected with software that is commercially available and are reported in the methods. Voluntary wheel running was monitored via an 86110 Sensor/Counter plugged into a central interface connected to a dedicated PC with Scurry Activity Monitoring Software (Lafayette Instrument, model 86165). Object location memory and elevated plus maze exploratory behavior was recorded and was analyzed through ANY-maze tracking software (version 4.99), (Stoelting Co.). Video Freeze software (version 3.0.0.0), (MedAssociates) automatically collected and scored freezing and motion index/locomotor activity for context fear conditioning experiments. LTP data were collected and digitized by NAC 2.0 Neurodata Acquisition System (Theta Burst).
Data analysis	Data were analyzed with open-source and commercially available code. Descriptions of analyses used are included in the methods. When appropriate, published descriptions of the methods with full references are included. Object location memory and elevated plus maze exploratory behavior was recorded and was analyzed through ANY-maze tracking software (version 4.99), (Stoelting Co.). Video Freeze software (MedAssociates) automatically scored freezing and motion index/locomotor activity for context fear conditioning experiments. Data were collected and digitized by NAC 2.0 Neurodata Acquisition System (Theta Burst). The RNA-seq data for each library were post-processed to produce FastQ files, then demultiplexed and filtered using both Illumina software CASAVA 1.8.2 as well as in-house software. Alignment to the reference genome and transcriptome: the reads from each replicate experiment were separately aligned to the reference genome and corresponding transcriptome using the short-read aligners ELAND v2e (Illumina) and Bowtie. Gene expression and differential analysis:FastQ files are processed through standard Tuxedo protocol outputting FPKM values for each gene of each replicate. Differential analysis of gene expression is conducted with Cyber-T, an analysis program using Bayesian-regularized t-test. Enrichment of each group for Gene Ontology terms, and KEGG pathways was assessed using DAVID, based on differentially expressed genes after learning. Data visualization was performed using 'matplotlib' for python and 'ggplot' for R. All other statistics were performed with GraphPad Prism 8 software.

For manuscripts utilizing custom algorithms or software that are central to the research but not yet described in published literature, software must be made available to editors and reviewers. We strongly encourage code deposition in a community repository (e.g. GitHub). See the Nature Portfolio guidelines for submitting code & software for further information.

Data

Policy information about availability of data

All manuscripts must include a data availability statement. This statement should provide the following information, where applicable:

- Accession codes, unique identifiers, or web links for publicly available datasets
- A description of any restrictions on data availability
- For clinical datasets or third party data, please ensure that the statement adheres to our policy

The raw RNA sequencing data have been deposited in NCBI's Gene Expression Omnibus (GEO) and are accessible through GEO Series accession number GSE208615 [https://www.ncbi.nlm.nih.gov/geo/query/acc.cgi?acc=GSE208615]. Source data are provided with this paper. Human brain transcriptome data were obtained from the publicly available Genotype-Tissue Expression (GTEx) project [https://www.gtexportal.org/home/downloads/adult-gtex/bulk_tissue_expression]. The paper does not report original code.

Research involving human participants, their data, or biological material

Policy information about studies with human participants or human data. See also policy information about sex, gender (identity/presentation), and sexual orientation and race, ethnicity and racism.

Reporting on sex and gender	N/A
Reporting on race, ethnicity, or other socially relevant groupings	N/A
Population characteristics	N/A
Recruitment	N/A
Ethics oversight	N/A

Note that full information on the approval of the study protocol must also be provided in the manuscript.

Field-specific reporting

Please select the one below that is the best fit for your research. If you are not sure, read the appropriate sections before making your selection.

☒ Life sciences ☐ Behavioural & social sciences ☐ Ecological, evolutionary & environmental sciences

For a reference copy of the document with all sections, see nature.com/documents/nr-reporting-summary-flat.pdf

Life sciences study design

All studies must disclose on these points even when the disclosure is negative.

Sample size	We used published data to determine an optimal n for our behavioral, electrophysiology, and RNA-Seq studies (Vogel-Ciernia et al., 2013; White et al., 2016; Kwapis et al., 2018). Sample size is reported in the legends and methods.
Data exclusions	In order to ensure that mice included in analyses adequately explored the objects, mice were excluded from behavioral studies if object exploration did not occur for a combined period of at least 2s on training day (3 or 10 minute training session) or a combined period of 3s on test day (5 minute training session). To ensure that test scores were attributed to how well mice learned rather than object preference alone, mice that had a preference for object location on training day (DI of +/- 20) were also excluded from the study. Grubbs test was used as a method to exclude behavioral outliers (with a P value threshold of 0.05).
Replication	Each experiment was replicated successfully in at least two independent groups.
Randomization	All mice/samples in this study were randomly assigned to experimental or control groups. All treatments were administered in an alternating manner. Control and experimental groups of mice were always run together in behavioral studies to ensure that cohorts of mice were counterbalanced.
Blinding	Allocation of animals receiving viral injections with each age group was randomly assigned. Experimenters were blinded to genotype/experimental group before and during all behavioral experiments. Experimenters were blinded to groups during scoring of behavioral data and tissue collection. For electrophysiology studies, mice within each group were randomly allocated following cessation of behavior.

Reporting for specific materials, systems and methods

We require information from authors about some types of materials, experimental systems and methods used in many studies. Here, indicate whether each material, system or method listed is relevant to your study. If you are not sure if a list item applies to your research, read the appropriate section before selecting a response.

Materials & experimental systems

n/a	Involved in the study
	☒ Antibodies
☒	☐ Eukaryotic cell lines
☒	☐ Palaeontology and archaeology
	☒ Animals and other organisms
☒	☐ Clinical data
☒	☐ Dual use research of concern
☒	☐ Plants

Methods

n/a	Involved in the study
☒	☐ ChIP-seq
☒	☐ Flow cytometry
☒	☐ MRI-based neuroimaging

Antibodies

Antibodies used

The following antibodies were used in this study: Immunohistochemistry: Flag (1:500, Cell Signaling, ab14793, clone #: D6W5B); goat anti-rabbit Atto 488 (1:500, Sigma, ab18772); Acvr1c (1:100, LS Bio, LS-C119149-50). Chromatin Immunoprecipitation: chromatin was immunoprecipitated overnight with 2ug of anti-H3K27Ac (Abcam ab4729), 10 ul of anti-H3K27Me3 (Cell Signaling #9733, clone #: C36B11), 2ug of anti-H3K9Me3 (Abcam ab176916, clone #: EPR16601) or 10ul of anti-H3k9Ac (Cell Signaling #9649S, clone #: C5B11) or an equivalent amount of anti-rabbit IgG (negative control, Abcam ab171870).

Validation

All antibodies were validated for the species and applications indicated by the manufacturer. Flag antibody Cell Signaling, ab14793, clone #: D6W5B is a monoclonal antibody produced by immunizing animals with a synthetic DYKDDDDK peptide. It is reactive with human, mouse, rat, hamster, monkey, virus, mink, chicken, D. melanogaster, xenopus, zebrafish, bovine, dog, pig, S. cerevisiae, c. elegans, horse, guinea pig, and rabbit. Validated for IF, IHC, WB, IP, ChIP, C&R, C&T, DB, eCLIP, and flow cytometry. Goat anti-rabbit Atto 488 Sigma, ab18772 is a polyclonal antibody produced in goat. It may be used to detect and quantitate the level of IgG in rabbit serum and biological fluids via fluorogenic immunochemical or immunohistochemical techniques. It may also be used as a secondary antibody in assays that use rabbit IgG as the primary antibody. Anti-Rabbit IgG has been used for immunocytochemistry applications at a dilution of 1:500 in neural precursor cells. ALK7 antibody LS-C119149 is an unconjugated rabbit polyclonal antibody to ALK7 (ACVR1C) (aa201-250) from human. It is reactive with human, mouse and rat. Validated for IF, IHC and Peptide-ELISA. Anti-H3K27Ac (Abcam ab4729) is a ChIP grade rabbit polyclonal antibody. It is reactive with human, mouse, rat and cow. Validated for IF/ICC, IHC-P, ChIP and PepArr. Anti-H3K27Me3 (Cell Signaling #9733, clone #: C36B11) is a ChIP grade Monoclonal antibody produced by immunizing animals with a synthetic peptide corresponding to the amino terminus of histone H3 in which Lys27 is tri-methylated. It is reactive with human, mouse, rat and monkey. Validated for IF, IHC, ChIP, WB, flow cytometry, C&R, and C&T. Anti-H3K9Me3 (Abcam ab176916, clone #: EPR16601) is a ChIP grade rabbit monoclonal [EPR16601] antibody to Histone H3 (tri methyl K9). It is reactive with human, mouse and rat. Validated for IF/ICC, IHC-P, ChIP, PepArr, ChIP-Seq, WB, dot blot and ChIC/C&R-seq. Anti-H3k9Ac (Cell Signaling #9649S, clone #: C5B11) is a ChIP grade monoclonal antibody is produced by immunizing animals with a synthetic peptide corresponding to the amino terminus of histone H3 in which Lys9 is acetylated. It is reactive with human, mouse, rat, monkey and zebrafish. Validated for IF, IHC, ChIP, WB, C&R, IP, flow cytometry, and C&T.

Animals and other research organisms

Policy information about studies involving animals; ARRIVE guidelines recommended for reporting animal research, and Sex and Gender in Research

Laboratory animals

Male, 12-week-old C57BL/6J mice and 18-month-old C57BL/6J mice (Jackson Laboratory) were individually housed under standard conditions (12 h:12 h light and dark cycle) and provided ad libitum access to food and water. 18-month-old 5xFAD mice were bred by the Transgenic Mouse Facility at UCI. 5xFAD hemizygous (B6.Cg-Tg(APPSwFlLon,PSEN1*M146L*L286V)6799Vas/Mmjax, Stock number 34848-JAX, MMRRC) and its wildtype littermates were produced by crossing or IVF procedures with C57BL/6J (Jackson Laboratory) females. 5xFAD mice were group housed with wildtype (C57BL/6J) littermates to ensure accurate genotype-specific effects on behavior and synaptic plasticity.

Wild animals

This study did not involve wild animals.

Reporting on sex

Behavioral and electrophysiology experiments involve male subjects, therefore, analyses involving Sex as a factor were not applied. Experiments examining Acvr1c expression utilize both female and male animals (Fig. 6A and 5H); therefore, Sex is included as a variable in these analyses.

Field-collected samples

The study did not involve samples collected from the field.

Ethics oversight

Experiments were conducted in accordance with the National Institutes of Health guidelines for animal care and use and were approved by the Institutional Animal Care and Use Committee of the University of California, Irvine.

Note that full information on the approval of the study protocol must also be provided in the manuscript.
